# Identification of key genes in membranous nephropathy and non-alcoholic fatty liver disease by bioinformatics and machine learning

**DOI:** 10.3389/fimmu.2025.1564288

**Published:** 2025-06-05

**Authors:** Jiachen Fan, Na Li, Yanfang Lu, Huixia Cao

**Affiliations:** ^1^ People’s Hospital of Zhengzhou University, Zhengzhou, Henan, China; ^2^ Henan Provincial Key Laboratory of Kidney Disease and Immunology, Henan Provincial Clinical Research Center for Kidney Disease, Henan Provincial People’s Hospital, Zhengzhou, Henan, China

**Keywords:** membranous nephropathy, non-alcoholic fatty liver disease, immune, bioinformatics, machine learning, single-cell RNA-seq

## Abstract

**Background:**

Chronic kidney disease (CKD) and non-alcoholic fatty liver disease (NAFLD) are closely associated. However, membranous nephropathy (MN), one of the causes of CKD, may contribute to NAFLD through abnormalities in lipid metabolism.

**Methods:**

93 patients diagnosed with MN by renal biopsy and admitted to Henan Provincial People’s Hospital between August 2021 and August 2022 were enrolled in this study. Patients were divided into two groups based on the presence or absence of NAFLD. Publicly available datasets related to NAFLD and MN were obtained from the Gene Expression Omnibus (GEO) database. Differentially expressed genes (DEGs) were identified, and weighted gene co-expression network analysis (WGCNA) was conducted to identify module genes. Gene Ontology (GO) and Kyoto Encyclopedia of Genes and Genomes (KEGG) enrichment analyses were performed. A protein-protein interaction (PPI) network was constructed, and key genes associated with both diseases were identified using Cytoscape software and machine learning algorithms. The correlation between immune cell infiltration and the two diseases was evaluated using the CIBERSORT algorithm. Finally, the key gene expression was validated using external datasets and immunohistochemistry (IHC).

**Results:**

Compared with the non-NAFLD group, patients in the NAFLD group had significantly higher body weight, hemoglobin levels, triglycerides, and complement C3 and C4 levels. Conversely, IgG levels were significantly lower in the NAFLD group. A total of 211 shared DEGs were identified between MN and NAFLD, including 175 upregulated and 36 downregulated genes. Enrichment analysis indicated that these genes were primarily involved in immune and inflammatory responses. PPI network analysis identified seven hub genes: *CSF1R*, *FCGR1G*, *FCGR3A*, *VAV1*, *SPI1*, *HCK*, and *CCR1*. Among them, *CSF1R* was identified as the key gene using a machine learning approach.

**Conclusion:**

This study suggests that CSF1R is a shared molecular of MN and NAFLD, which may serve as a potential therapeutic target for patients affected by both diseases.

## 1 Introduction

Membranous nephropathy (MN) is a glomerular disease characterized by proteinuria and hypoalbuminemia, often accompanied by hyperlipidemia. The pathology of MN is the deposition of immune complexes in the glomerular basement membrane, leading to its thickening (1). MN is the most common pathological type of nephrotic syndrome (NS) in adults. Patients with MN not only face an increased risk of progression to end-stage renal disease (ESRD) compared to healthy individuals but are also at higher risk for complications such as heart failure, ischemic stroke, and venous thromboembolism (2, 3).

Non-alcoholic fatty liver disease (NAFLD) is becoming more commonplace due to social progress and lifestyle changes, with a current global prevalence rate estimated at 25% (4). NAFLD is characterized by excessive fat accumulation in the liver, excluding other causes of hepatic steatosis such as viral hepatitis, autoimmune hepatitis, and drug-induced liver injury (5). It encompasses a spectrum of conditions including non-alcoholic fatty liver (NAFL), non-alcoholic steatohepatitis (NASH), and NASH-related cirrhosis (6, 7).

Disorders of lipid metabolism are considered a key factor linking MN and NALFD. Due to the damage of the renal filtration barrier, a large amount of protein is lost from the urine. Hypoalbuminemia stimulates the liver to synthesize lipoproteins such as VLDL, IDL and LDL, thereby triggering hyperlipidemia (8). Patients with MN also have a reduction in both hepatic lipase activity, resulting in an increase in the level of free fatty acids (FFAs) in the circulation (9). As a result, the increased uptake of FFA by the liver directly promotes steatosis (10). In addition, patients with MN often present with insulin resistance, which is a central cause of NAFLD (11, 12). In the state of insulin resistance, insulin’s ability to suppress lipolysis in peripheral adipose tissue is impaired, leading to increased levels of FFAs in the circulation and excessive accumulation in the liver (13).

MN is a known cause of chronic kidney disease (CKD), and previous studies have shown that the prevalence of NAFLD increases with the progression of CKD (2, 14). Although multiple studies have reported an association between NAFLD and CKD, direct evidence linking MN to NAFLD is lacking. Furthermore, after adjusting for common risk factors, NS has been identified as a risk factor for NAFLD (15). However, the underlying molecular mechanisms and biological processes that drive this association remain poorly understood.

**Figure 1:**
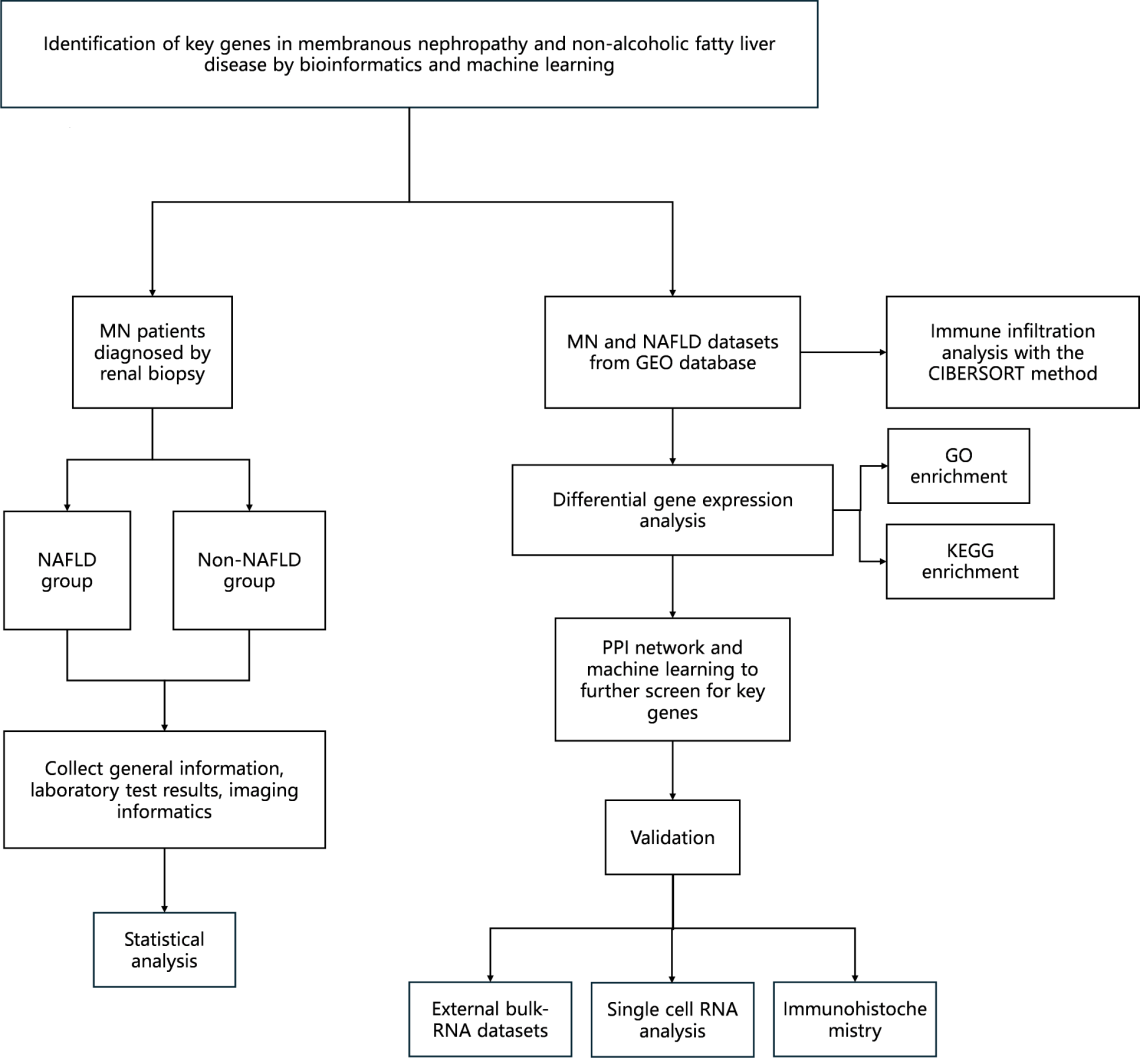
Technology roadmap constructed for this study.

Therefore, this study aims to explore the shared pathogenic mechanisms between MN and NAFLD using a bioinformatics approach. Notably, this is the first study to identify common molecular pathways and genetic signatures associated with both diseases, which is expected to provide a foundation for improved clinical management of patients affected by both conditions. The workflow of the study is illustrated in **Figure 1**.

## 2 Materials and methods

### 2.1 Patients

This study included 93 patients diagnosed with MN who were admitted to the Department of Nephrology of Henan Provincial People’s Hospital between August 2021 and August 2022. The inclusion and exclusion criteria for both MN and NAFLD are described below.

#### 2.1.1 MN

Inclusion criteria: age ≥ 18 years; pathological diagnosis of MN confirmed by renal biopsy; availability of complete clinical data.

Exclusion criteria: Presence of other glomerular diseases, such as IgA nephropathy, diabetic nephropathy, etc.; Secondary MN caused by malignancies, medications or autoimmune diseases; pregnancy or lactation; chronic kidney disease (CKD) stage 5 at the time of first diagnosis, or renal replacement therapy (dialysis or kidney transplantation).

#### 2.1.2 NAFLD

Inclusion criteria: age ≥ 18 years; liver steatosis diagnosed by abdominal ultrasonography.

Exclusion criteria: heavy alcohol consumption (>210 g/week for men and >140 g/week for women); viral hepatitis, liver disease caused by drugs, autoimmune liver disease or other specific diseases known to cause fatty liver.

### 2.2 Inclusion index

Baseline patient data (gender, age, height, weight, blood pressure, alcohol consumption history), routine blood examinations (hemoglobin[Hb], white blood cells [WBC], platelets [PLT]), biochemical indicators (alanine aminotransferase [ALT], aspartate aminotransferase [AST], albumin [ALB], cholesterol [CHOL], TG, HDL, LDL, blood uric acid [UA], glucose [GLU], blood urea nitrogen [BUN], serum creatinine [Scr], cystatin C [CysC], 24-h urine protein, phospholipase A2 receptor antibody [PLA2R], complement C3, complement C4, immunoglobulin G [IgG], immunoglobulin M[IgM], immunoglobulin A[IgA] and D-Dimer), infectious diseases biomarkers (hepatitis B surface antigen [HBsAg], hepatitis C antibody [anti-HCV]) were collected at the time of first diagnosis of MN. This study was approved by the Medical Ethics Committee of Henan People’s Hospital (approval number: 2020207), and informed consent was obtained from all participants.

A *post hoc* power analysis was conducted to assess the statistical power of the study based on observed differences in key clinical variables. The analysis revealed that the sample size of 93 patients provided sufficient power (>80%) for detecting clinically significant differences. Specifically, the power exceeded 88% for all major variables, including body weight (power = 88%), triglyceride levels (power = 96%), 24-hour urine protein excretion (power = 98%), C3 levels (power = 88%), and C4 levels (power = 99%). These results confirm that the current sample size was adequate to detect the observed differences with a significant level of 0.05.

### 2.3 Datasets collection and preprocessing

Gene expression profiles of numerous diseases can be found in the Gene Expression Omnibus (GEO), a publicly available genomics data repository. Using “Membranous nephropathy” and “Nonalcoholic fatty liver disease” as keywords, 6 datasets were downloaded including GSE126848 (16), GSE197307 (17), GSE89632 (18), GSE104948 (19), GSE185051 (20) and GSE200828. Among them, GSE126848 and GSE197307 were used for differential expression analysis, while GSE89632, GSE104948, GSE185051, and GSE200828 served as validation datasets (**Table 1**).

**Table 1:** Datasets used for this study.

Datasets	Platforms	Disease	Samples	Group
GSE126848	GPL18573	NAFLD	15 patients and 14 controls	Discovery
GSE197307	GPL18573	MN	62 patients and 8 controls	Discovery
GSE89632	GPL14951	NAFLD	19 patients and 24 controls	Validation
GSE185051	GPL24676	NAFLD	52 patients and 5 controls	Validation
GSE200828	GPL19983	MN	51 patients and 6 controls	Validation
GSE104948	GPL24120, GPL22945	MN	21 patients and 21 controls	Validation

Quality control was conducted using the pheatmap package in R to assess the correlation of gene expression among samples (**Supplementary Figure 1A, D**). Samples with low correlation coefficients were considered outliers and removed. The remaining samples were further assessed using principal component analysis (PCA) to evaluate clustering and detect hidden confounding factors (**Supplementary Figure 1B, C, E-F**). When a gene mapped to multiple probe IDs, the mean expression levels of the same symbol were considered as the final gene-level expression.

### 2.4 Weighted gene co-expression network analysis in NAFLD patients

The WGCNA package in R was used to construct gene co-expression networks and identify gene modules (21). First, the goodSamplesGenes function was used to filter out low-quality genes and samples. The soft threshold was determined using the pickSoftThreshold function, selecting the smallest β where the scale-free topology fit index exceeded 0.85. Subsequently, the blockwiseModules function was utilized to identify the gene module, with minModuleSize set to 30 and MEDissThres set to 0.25. Genes with similar expression profiles were grouped into the same module. Finally, the module eigengenes (ME) were calculated by using the moduleEigengenes function. Correlations between MEs and clinical traits were then evaluated to identify modules most associated with disease. Genes from these trait-associated modules were selected for further analysis based on their module membership (MM) and gene significance (GS).

### 2.5 Identification of DEGs of MN and NAFLD

DEGs between disease and control groups were identified using the DESeq2 package in R (22). The screening thresholds were set as |log2FoldChange| > 1 and adjusted p-value < 0.05 (**Supplementary Table 2**). Genes with log2FC > 1 were considered up-regulated, whilst those with log2FC < -1 were considered downregulated. Heatmaps and volcano plots were generated using the pheatmap and ggplot2 packages, respectively (23). To improve robustness, only the overlapping DEGs from both GSE126848 and GSE197307 were retained for downstream analysis. Shared genes were defined as the intersection between DEGs and trait-related module genes identified by WGCNA, determined using the ggvenn package.

### 2.6 Enrichment analysis of shared genes

Functional enrichment of the shared genes was performed using the clusterProfiler package in R. Both Gene Ontology (GO) and Kyoto Encyclopedia of Genes and Genomes (KEGG) pathway analyses were conducted, with a significance threshold set at p < 0.05 (24).

### 2.7 Protein-protein interaction network establishment and hub-gene identification

Shared genes were submitted to the STRING database (http://string-db.org/) to construct a high-confidence PPI network (minimum required interaction score ≥ 0.700). The network was visualized and analyzed using Cytoscape software (v3.10.2). The cytoHubba plugin (v0.1) was employed to rank nodes based on four algorithms: Radiality, Maximum Neighborhood Component (MNC), Maximal Clique Centrality (MCC), and Degree. Hub genes that were consistently ranked among the top 10 across algorithms were retained.

To further explore the diagnostic potential of these hub genes, a random forest (RF) model was constructed using gene expression data from the NAFLD datasets. The number of trees (ntree) was tuned to achieve the best performance, and the mtry parameter was set to the square root of input features. The final model was used to identify key diagnostic genes with maximum specificity and sensitivity. Model performance was assessed using the area under the receiver operating characteristic (ROC) curve in the training sets and subsequently validated on the test datasets.

### 2.8 Validation of the key gene

The expression level of the identified key gene was evaluated across four independent validation datasets (GSE89632, GSE104948, GSE185051 and GSE200828). Differences between disease and control samples were visualized using box plots.

### 2.9 Immune infiltration analysis

To assess the immune microenvironment in both MN and NAFLD, immune cell proportions were estimated using the CIBERSORT algorithm, which deconvolutes bulk expression data into 22 immune cell types. The results were visualized using the ggplot2 package. Pearson correlation analysis was performed to examine the association between the key gene and immune cell infiltration levels, and results were displayed as heatmaps.

### 2.10 Human biopsy specimens and immunostaining procedures

All patients with MN were confirmed through pathological examination. Healthy kidney tissues adjacent to malignant lesions were obtained during nephrectomy for kidney cancer. Liver tissues were collected from patients undergoing hepatic surgery for conditions such as hepatic hemangiomas or liver cysts. The experimental procedures conducted in this study received approval from the Henan People’s Hospital Ethics Committee (approval number: 2020207). Paraffin-embedded kidney and liver tissues were cut into 5-µm-thick slices. The deparaffinized slices were subjected to antigen retrieval and endogenous peroxidase inactivation. The slides were then blocked with 3% bovine serum albumin (BSA) at room temperature for 30 minutes. According to the manufacturer’s instructions, sections were incubated overnight at 4°C with anti-CSF1R antibodies (Proteintech, Cat# 25949-1-AP, RRID: AB_2880306). After incubation, slices were washed thrice with phosphate-buffered saline (PBS), incubated with the appropriate secondary antibodies (HRP- labeled goat anti-rabbit IgG) (ServiceBio Cat# GB23303, RRID: AB_2811189) at 37 °C for 50 mins. The slides were then washed again with PBS. Color development was performed using 3,3′-diaminobenzidine (DAB) and examined under a light microscope. Then use Lignin to stain for 3 mins.

Tissue sections were deparaffinized and antigen retrieval. After cooling, slides were washed with PBS, and non-specific binding was blocked using 3% BSA for 30 minutes at room temperature. Sections were incubated with anti-CSF1R antibodies (Proteintech, Cat# 25949-1-AP, RRID: AB_2880306) overnight at 4 °C in a humidified chamber, followed by PBS washes and incubation with fluorescent secondary antibodies (ServiceBio Cat# GB21303, RRID: AB_2861435) for 50 minutes at room temperature in the dark. Nuclei were counterstained with DAPI, and autofluorescence was quenched before mounting with anti-fade medium. Fluorescence was visualized using a fluorescence microscope with the following filters: DAPI (Ex 330–380 nm/Em 420 nm, blue) and CY3 (Ex 510–560 nm/Em 590 nm, red).

Quantitative analysis was carried out using ImageJ software by comparing the experimental groups to the control group.

### 2.11 Single cell sequencing

Due to the absence of human single-cell RNA sequencing (scRNA-seq) data for NAFLD, the mouse dataset GSE129516 was selected for validation. Two human MN datasets, GSE241302 and GSE131685, were also obtained for analysis. All datasets were downloaded from the GEO database and processed using the Seurat R package (25). Cells expressing less than 200 genes and genes expressed in fewer than 3 cells were filtered out. Doublet cells were identified and excluded from all samples. Ambient RNA contamination was estimated and removed. Raw gene expression counts were normalized using the LogNormalize method, scaling each cell’s expression by a factor of 10,000. Subsequently, variance stabilization transformation (VST) was applied to identify the top 2,000 highly variable genes per sample. These genes were then scaled using the ScaleData function. Dimensionality reduction was performed using principal component analysis (PCA) via the RunPCA function. All samples were integrated using the Harmony algorithm to correct for batch effects. For MN datasets (26), the top 11 principal components (PCs) were selected for cell clustering using the FindNeighbors and FindClusters functions. For the NAFLD dataset, the top 10 PCs were used. Uniform Manifold Approximation and Projection (UMAP) was applied for visualization using the RunUMAP function. Cluster-specific marker genes were identified using the FindAllMarkers function, based on the Wilcoxon rank-sum test.

### 2.12 Statistical analysis

Statistical analyses were performed using SPSS software (version 27.0) and R software (version 4.4.1). For continuous variables, the t-test was used to compare normally distributed data, while the Mann–Whitney U test was applied for non-normally distributed data. Categorical variables were expressed as percentages and compared using Pearson’s Chi-square test or Fisher’s exact test, as appropriate. A two-sided *P* value of <0.05 was considered statistically significant.

## 3 Results

### 3.1 Baseline data

From August 2021 to August 2022, a total of 93 patients diagnosed with MN were enrolled in this study. These patients were divided into two groups based on the presence or absence of NAFLD. A comparison of baseline characteristics between the NAFLD and non-NAFLD groups is presented in **Supplementary Table 1**. Patients in the NAFLD group had significantly higher body weight (75.02 vs. 66.69 kg, *P* < 0.001), triglyceride (TG) levels (2.68 vs. 1.95 mmol/L, *P* = 0.001), and 24-hour urinary protein excretion (6.94 vs. 3.78 g/L, *P* = 0.002) compared to those in the non-NAFLD group. Complement levels were also significantly elevated in the NAFLD group, with higher C3 (1.32 vs. 1.17 g/L, *P* < 0.001) and C4 (0.36 vs. 0.29 g/L, *P* = 0.003). No significant differences were observed between the two groups in terms of sex, age, height, systolic blood pressure (SBP), diastolic blood pressure (DBP), white blood cell count (WBC), hemoglobin (Hb), platelet count (PLT), AST, ALT, albumin (ALB), cholesterol (CHOL), HDL, LDL, blood urea nitrogen (BUN), serum creatinine (Scr), uric acid (UA), glucose (GLU), cystatin C (CysC), anti-PLA2R, IgA, IgM and D-dimer levels.

### 3.2 Selection of module genes by WGCNA

**Figure 2:**
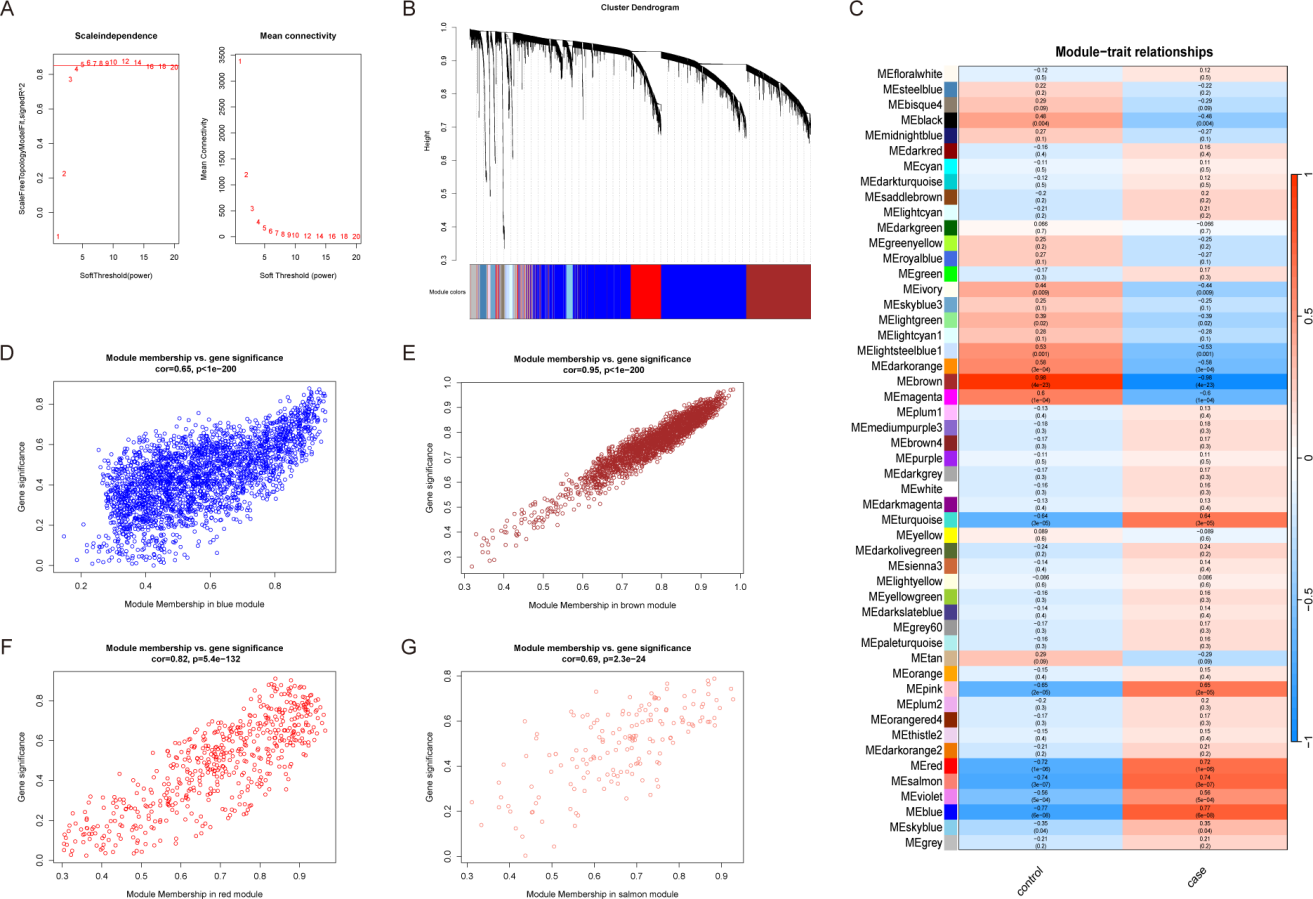
Weighted gene co-expression network analysis. **(A)** Selection of the best soft threshold value. The optimal soft threshold is when the scale-free fit index first approaches the red line (represents 0.85). **(B)** Similar genes are clustered together in the Cluster dendrogram, with the top of the image representing the clustering dendrogram and the bottom representing the color corresponding to each gene. **(C)** Heatmap of relationships between module genes and clinical traits. Red, blue and salmon module genes had a high positive correlation with NAFLD. Brown module genes were negatively and strongly correlated with NAFLD. **(D–G)** Module significance and gene significance of red, blue, salmon and brown module genes were positively correlated.

A weighted gene co-expression network was constructed using the expression data of 17,249 genes from 26 NAFLD patients and 9 healthy controls. A soft-thresholding power of β = 5 was selected to achieve a scale-free topology (**Figure 2A**). A total of 33 distinct gene modules were identified. Among them, the yellow, brown, red, and salmon modules exhibited the strongest correlations with NAFLD (**Figures 2B–C**). The relationships between module membership (MM) and gene significance (GS) for these key modules are illustrated in **Figures 2D–G**.

### 3.3 Identification of DEGs in MN and NAFLD

**Figure 3:**
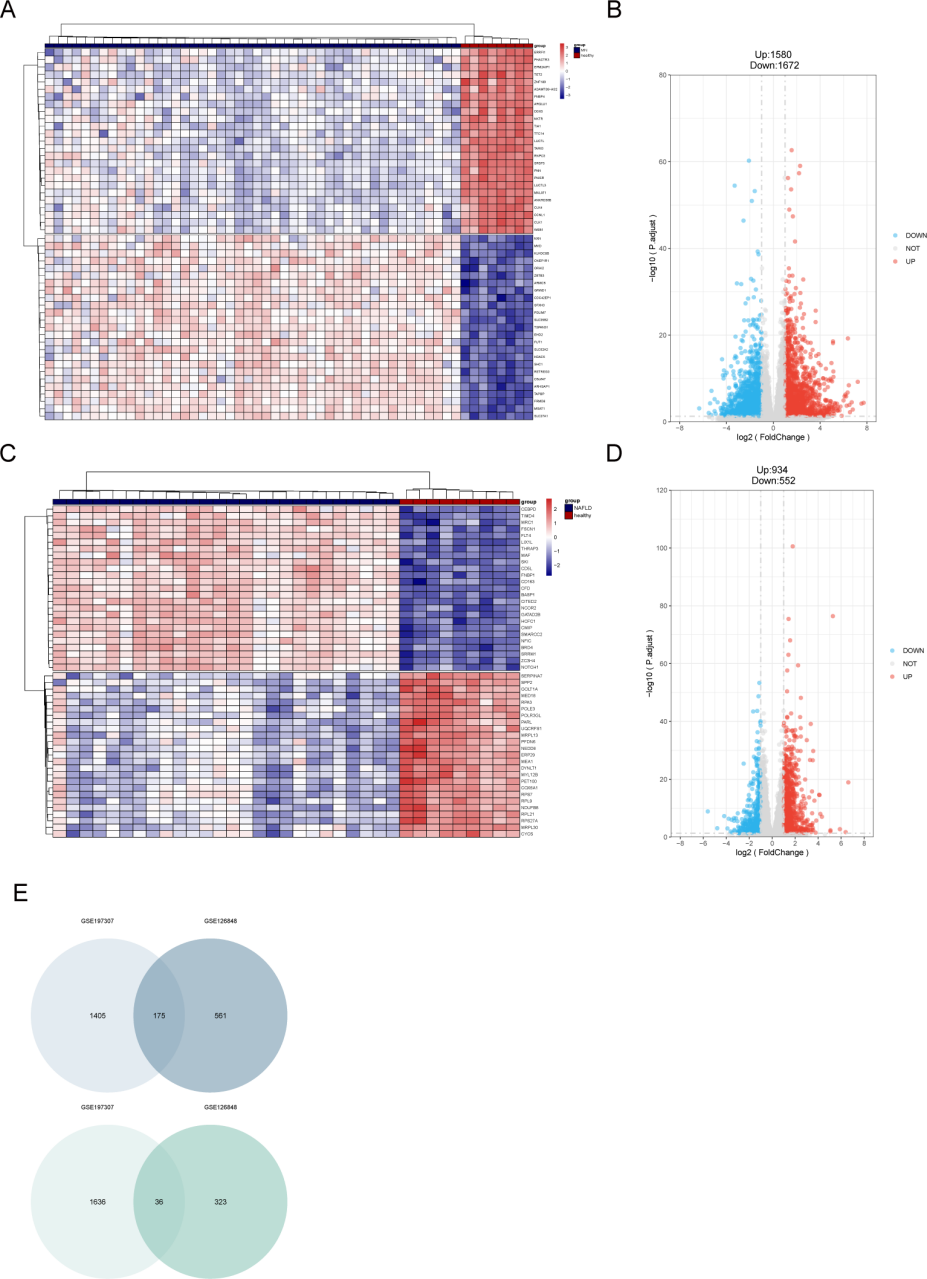
Identification of DEGs of MN and NAFLD. **(A)** Heatmap showing the top 100 DEGs in the MN dataset according to the padj value. **(B)** Volcano plot of DEGs of MN, including 1580 upregulated genes and 1672 downregulated genes. **(C)** Heatmap showing the top 100 DEGs in the NAFLD dataset according to the padj value. **(D)** Volcano plot of DEGs of NAFLD, containing 934 upregulated genes and 552 downregulated genes. **(E)** Venn plot of common genes. DEGs, differentially expressed genes; MN, membranous nephropathy; NAFLD, non-alcoholic fatty liver disease; padj: adjusted *P* value.

In the GSE197307 dataset, a total of 3,252 differentially expressed genes (DEGs) were identified, including 1,580 upregulated and 1,672 downregulated genes. In the NAFLD dataset, 934 genes were significantly upregulated, while 552 genes were significantly downregulated. Volcano plots display the distribution of DEGs using different colors (**Figures 3B, D**), and the top 50 upregulated and downregulated genes are visualized in heatmaps (**Figures 3A, C**). Genes with consistent expression trends across both MN and NAFLD datasets were identified, and their intersection is illustrated in **Figure 3E**.

### 3.4 Functional enrichment analysis

**Figure 4:**
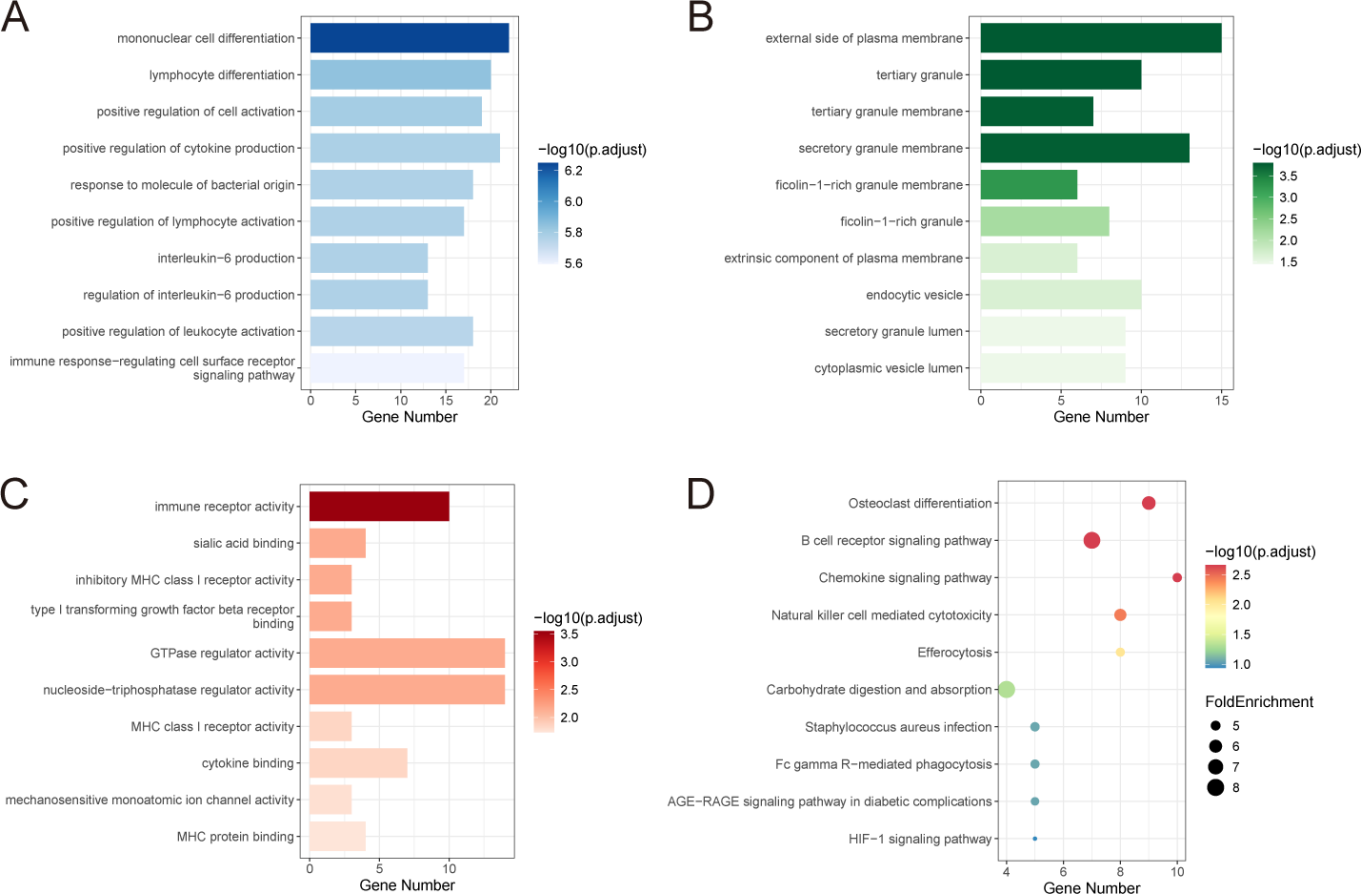
Functional enrichment analysis of shared genes. **(A-C)** GO enrichment analysis of shared genes, including biological process **(A)**, cellular component **(B)** and molecular function **(C)**. **(D)** KEGG enrichment of shared genes. FoldEnrichment revealed differences between the pathway’s proportions in this dataset and the entire genome. GO, Gene Ontology; KEGG, Kyoto Encyclopedia of Genes and Genomes.

To explore the biological functions of the upregulated and downregulated genes, GO and KEGG enrichment analyses were performed. GO analysis categorized gene functions into three domains: biological process (BP), cellular component (CC), and molecular function (MF). The most significantly enriched BP terms included positive regulation of cytokine production, leukocyte activation involved in immune response, cell activation involved in mononuclear cell differentiation, lymphocyte differentiation, and positive regulation of cell activation (**Figure 4A**). For CC, genes were primarily enriched on the external side of the plasma membrane, tertiary granule, and tertiary granule membrane (**Figure 4B**). In the MF category, enriched terms included immune receptor activity, inhibitory MHC class I receptor activity, sialic acid binding, and cytokine binding (**Figure 4C**). KEGG pathway analysis indicated that the shared DEGs were mainly involved in immune-related pathways, such as the B cell receptor signaling pathway, chemokine signaling pathway, and natural killer cell-mediated cytotoxicity (**Figure 4D**).

### 3.5 Establishment of PPI network

**Figure 5:**
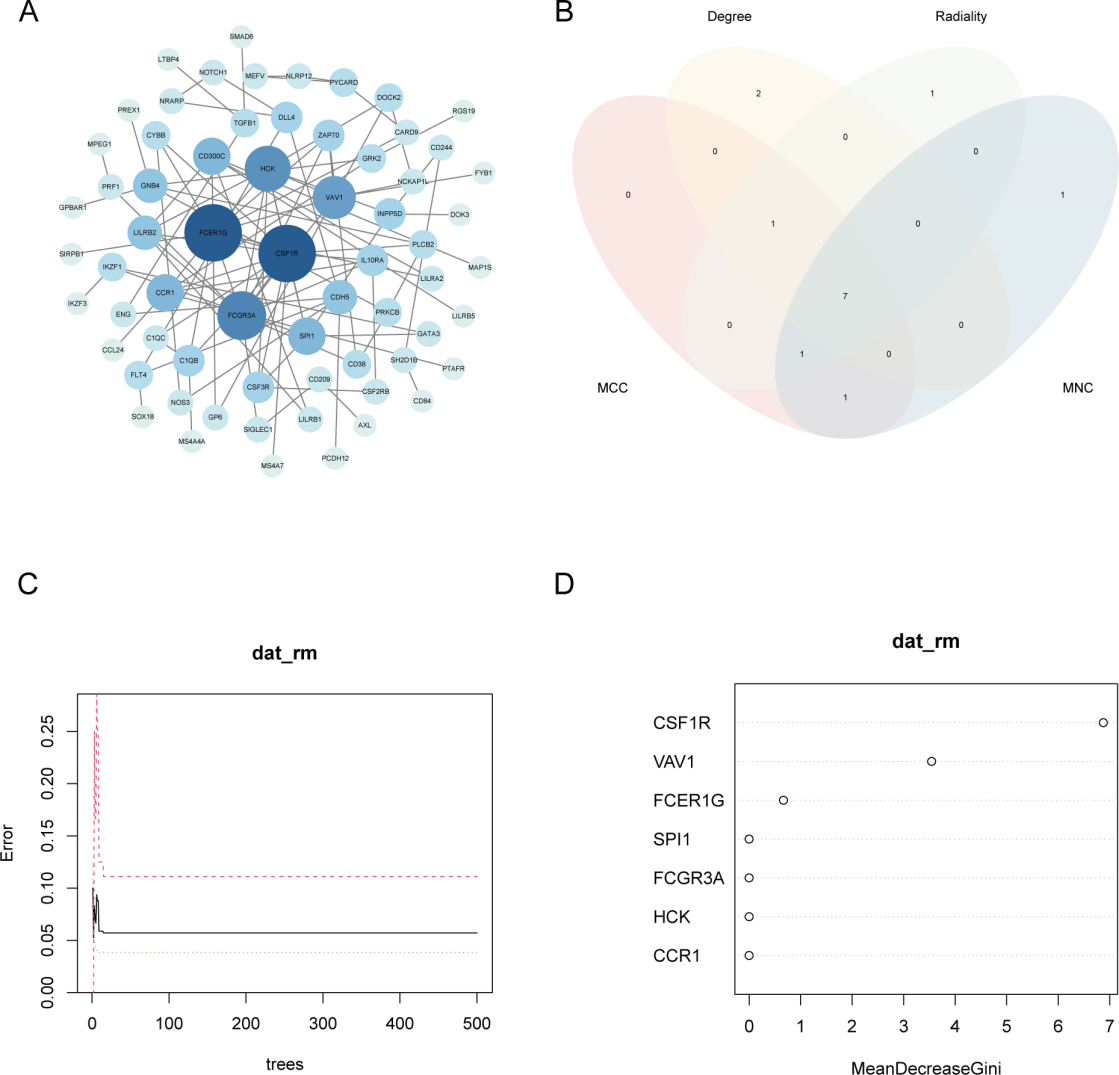
Gene selection by the PPI network and machine learning. **(A)** The PPI network of common genes. Different colors represent the connectivity of the gene. The darker the color, the more core the gene is in the network. **(B)** Venn diagram of the top 20 genes with the highest scores for 4 algorithms. **(C)** Relationship between the overall error rate and the number of trees. **(D)** Relative importance of each gene in the Random Forest model. CSF1R had the highest MeanDecreaseGini. PPI, protein-protein interaction.

To further explore the potential interactions among proteins encoded by the shared DEGs, a protein-protein interaction (PPI) network was constructed using the STRING database, with *Homo sapiens* set as the species. The resulting network, consisting of 210 nodes and 106 edges (interaction score > 0.7), was visualized using Cytoscape software (**Figure 5D**). To identify key hub genes, the cytoHubba plugin was employed (27). The top 20 genes from each method were intersected to identify common hub genes (**Figure 5B**). This analysis yielded seven hub genes: *VAV1*, *CSF1R*, *FCER1G*, *FCGR3A*, *CCR1*, *HCK*, and *SPI1*. The biological functions of these genes are summarized in **Table 2**.

**Table 2:** Proteins encoded by hub genes and their functions.

Gene	Protein	Function
VAV1	Vav guanine nucleotide exchange factor 1	A signaling molecules is involved in activating the Rho GTPase family, T cell receptor signaling (including Calcium flux, EARK signaling pathway, and Dynamin2) and regulating the cytoskeleton (61)
CSF1R	Colony stimulating factor 1 receptor	A cell-surface receptor playing an important role in the survival, proliferation and differentiation of macrophages and monocytes (62)
HCK	HCK proto-oncogene	A tyrosine-protein kinase which is involved in extracellular signals transmission, cell migration, cell differentiation and cell proliferation (63)
FCER1G	Fc epsilon receptor Ig	A component of the Fc portion of immunoglobulin E receptor playing an important role in mediating allergic inflammatory signaling in mast cells (64)
FCGR3A	Fc gamma receptor IIIa	A component of the Fc portion of immunoglobulin G receptor, which is involved in antibody-dependent cell-mediated cytotoxicity (ADCC) and antibody-dependent cell phagocytosis (ADCP) (65)
SPI1	Spi-1 Proto-Oncogene	A member of the transcription factor family Ets, which is involved in myeloid and B cells differentiation (66)
CCR1	CC chemokine receptor 1	A chemokine receptor that plays a critical role in the recruitment and activation of macrophages and monocytes at sites of inflammation (67)

### 3.6 Further selection of key genes

**Figure 6:**
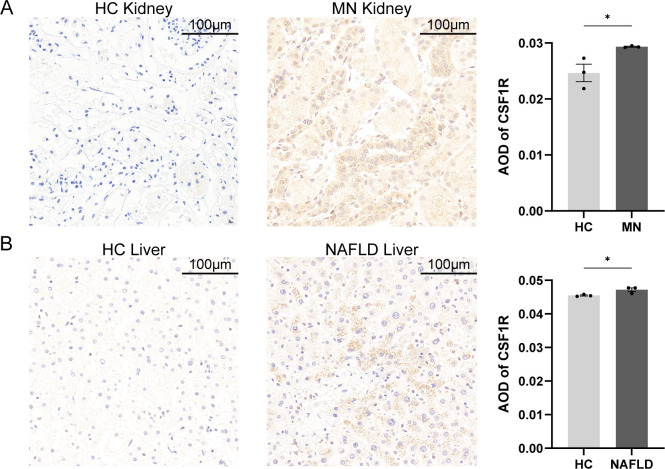
Histopathology of tissues from patients with MN, patients with MN and healthy individuals. **(A)** Kidney biopsies of MN patients and healthy controls. **(B)** Liver biopsies of NAFLD patients and healthy controls. Scalebars, 100μm. Data were presented as mean ± SEM. MN, membranous nephropathy; NAFLD, non-alcoholic fatty liver disease; AOD, average optical density, *P < 0.05.

To further identify the gene most closely associated with both MN and NAFLD, a random forest (RF) algorithm was applied to the seven hub genes. In this model, gene expression levels served as independent variables, while disease status was used as the dependent variable. Genes were ranked based on the MeanDecreaseGini index to determine their relative importance. The number of trees (ntree) was initially set to 500; as the number of trees increased, the model error stabilized. The optimal parameter was identified as ntree = 2, corresponding to the lowest model error (**Figure 5C**). The model exhibited strong discriminatory power, with area under the curve (AUC) values exceeding 0.9 in both the training and validation datasets, indicating excellent predictive performance (**Supplementary Figure 2**). Among the variables evaluated by the RF model, CSF1R demonstrated the highest MeanDecreaseGini value and was therefore identified as the most important gene (**Figure 5D**). To validate the expression of CSF1R, immunohistochemical staining was performed on liver and kidney biopsy tissues obtained from healthy controls, NAFLD patients, and MN patients (**Figure 6**). Results showed that CSF1R expression was minimal in healthy individuals, but moderately elevated in the renal vascular endothelium of MN patients and in hepatocytes of NAFLD patients. Similarly, compared with the control group, the disease groups showed an increase in CSF1R fluorescence intensity (**Supplementary Figure 3**). These findings suggest that immune dysregulation, particularly the upregulation of CSF1R, may underlie the pathophysiological link between MN and NAFLD.

### 3.7 Infiltration of Immune cells in NAFLD and MN

**Figure 7:**
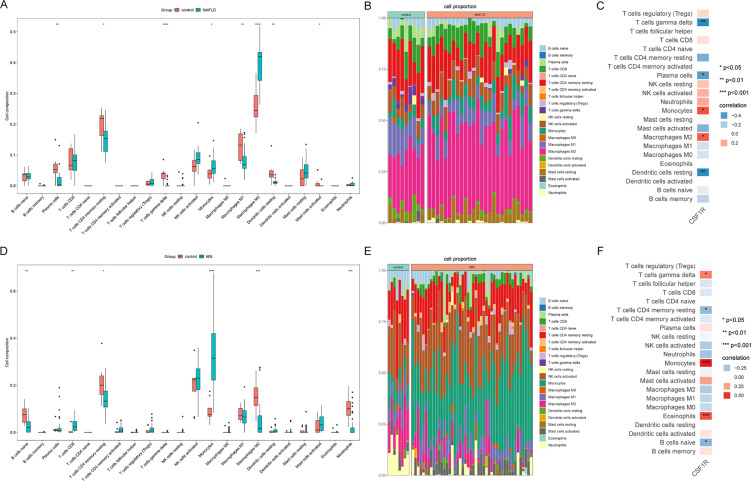
Immune infiltration analysis of MN and NAFLD. **(A)** Comparison of renal immune infiltration between healthy control and NAFLD patients. **(B)** Proportional composition of immune cells in each sample of NAFLD. **(C)** Correlation between CSF1R expression and immune cells in the NAFLD dataset. **(D)** Comparison of renal immune infiltration between healthy control and MN patients. **(E)** Proportional composition of immune cells in each sample of MN. **(F)** Correlation between CSF1R expression and immune cells in the MN dataset. MN, membranous nephropathy; NAFLD, non-alcoholic fatty liver disease; *P < 0.05; **P < 0.01; ***P < 0.001; ****P<0.0001.

Based on the findings from enrichment analyses and machine learning, immune dysregulation may serve as a potential link between MN and NAFLD. To further explore this hypothesis, the CIBERSORT algorithm was employed to assess immune cell infiltration in both diseases. The proportions of 22 immune cell subtypes in MN and NAFLD samples are depicted in bar plots (**Figure 7B, E**). Compared to healthy controls, both disease groups exhibited a significant increase in monocyte infiltration (**Figure 7A, D**), accompanied by a marked decrease in resting memory CD4^+^ T cells. To assess the relationship between immune cell composition and CSF1R expression, Pearson correlation analysis was conducted. In MN samples, CSF1R expression showed a strong positive correlation with M2 macrophages and monocytes, and a negative correlation with γδ T cells, plasma cells, and resting dendritic cells (**Figure 7C**). Similarly, in NAFLD, CSF1R expression was positively associated with γδ T cells, monocytes, and eosinophils, while inversely correlated with resting memory CD4^+^ T cells and naive B cells (**Figure 7F**).

### 3.8 Single cell sequencing analysis

**Figure 8:**
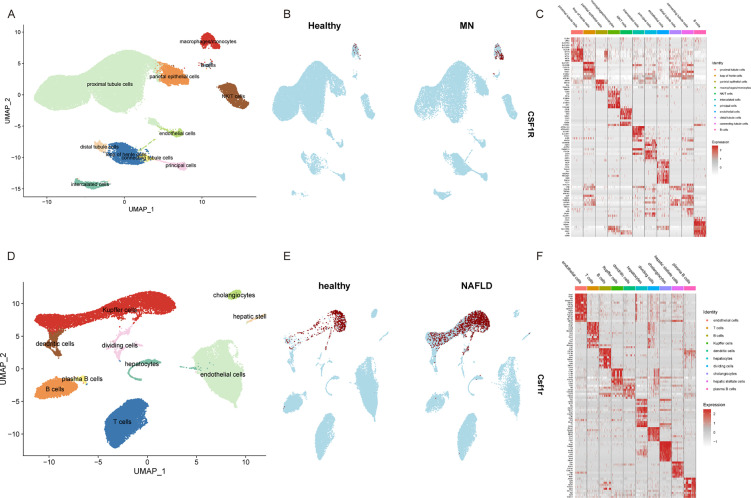
Single cell sequencing analysis for validation. **(A)** All samples from GSE241302 and GSE131685 were integrated and visualized using UMAP. **(B)** UMAP visualization of CSF1R expression in healthy individuals and MN patients. **(C)** Heatmap showed the expression of representative marker genes for each cell type. **(D)** All samples from GSE129516 were integrated and visualized using UMAP. **(E)** UMAP visualization of Csf1r expression in healthy individuals and NAFLD mice. **(F)** Heatmap showed the expression of representative marker genes for each cell type. MN, membranous nephropathy; NAFLD, non-alcoholic fatty liver disease; UMAP, Uniform Manifold Approximation and Projection.

In addition to transcriptomics analysis, single cell RNA sequencing was conducted to further validate our findings. After quality control, a total of 40864 integrated kidney cells were divided into 18 clusters. Based on marker genes documented in the literature, these clusters were annotated into 11 cell types, including proximal tubule cells, natural killer T cell (NK/T) cells, parietal epithelial cells, loop of Henle cells, intercalated cells, distal tubule cells, monocytes, distal tubule-immune cells, B cells, endothelial cells and principal cells. As **Figure 8A, D** showed, UMAP was utilized to visualize the cell cluster. Similarly, 30632 liver cells were divided into 16 clusters, which were further classified into 10 types. Marker genes for each cell type are illustrated in **Figure 8C, F**.

**Figure 9:**
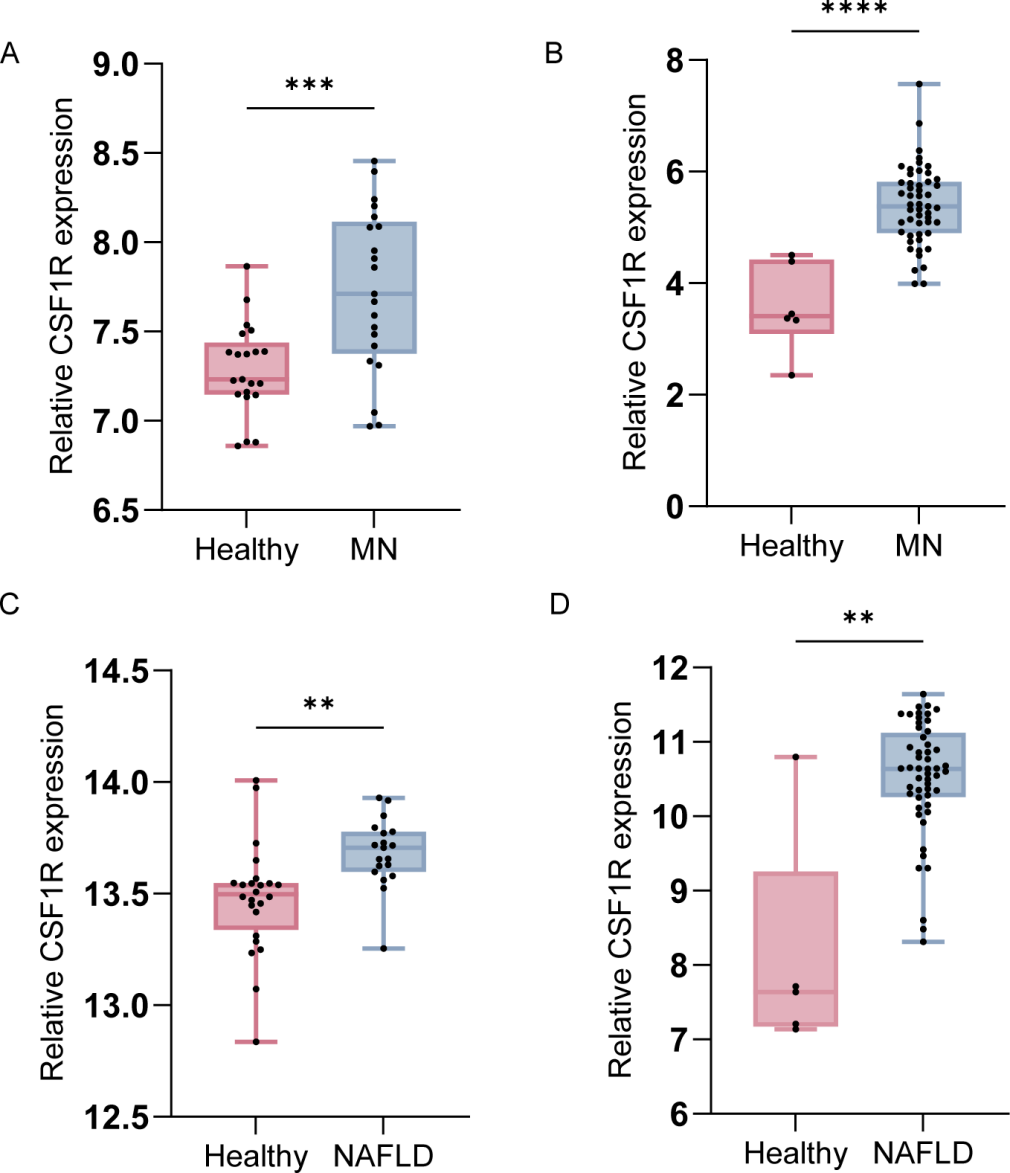
Boxplots of CSF1R expression in external datasets for validation. **(A)** Comparison of CSF1R expression between healthy individuals and MN patients in GSE104948. **(B)** Comparison of CSF1R expression between healthy individuals and MN patients in GSE200828. **(C)** Comparison of CSF1R expression between healthy individuals and NAFLD patients in GSE GSE89632. **(D)** Comparison of CSF1R expression between healthy individuals and NAFLD patients in GSE185051. Data were presented as mean ± SEM. MN, membranous nephropathy; NAFLD, non-alcoholic fatty liver disease; **, *P* < 0.01; ***, *P* < 0.001; ****, *P* <0.0001.

Box plots (**Figure 9**) showed that significantly elevated expression of CSF1R in disease groups compared to healthy controls in external validation datasets. Additionally, CSF1R expression was elevated in both MN and NAFLD at the single cell level (**Figure 8B, E**). Notably, CSF1R was predominantly enriched in the mononuclear phagocyte system, aligning with findings from the immune infiltration analysis. These results suggest that monocytes may play a pivotal role in the pathogenesis of both NAFLD and MN.

## 4 Discussion

It is well established that NAFLD and CKD are closely linked, yet the specific CKD subtypes associated with NAFLD remain unclear. NS, particularly MN, is characterized by significant lipid metabolism disorders, which may contribute to NAFLD development (15, 28). In this study, we explored the clinical features and molecular mechanisms of MN patients with NAFLD through the synergistic integration of bioinformatics analysis and machine learning approaches.

Nearly 40% of MN patients in our cohort had NAFLD. These patients exhibited more severe proteinuria, suggesting a worse renal prognosis (29). They also had lower IgG levels, consistent with prior reports (30). However, some studies showed high IgG levels may promote NAFLD development and increase the risk of hepatic decompensation (31, 32). Additionally, we observed altered complement activity, including elevated serum C3 levels, which supports previous findings of complement activation in NAFLD (33, 34). In conclusion, we hypothesized that the occurrence of NAFLD in MN patients may be associated with immune disorders and exacerbate pre-existing diseases.

In addition, lipid metabolism may represent a mechanistic link between MN and NAFLD. Elevated levels of FFAs promote mitochondrial β-oxidation, leading to the overproduction of reactive oxygen species (ROS) and hepatocyte apoptosis (35). Similarly, FFAs impair mitochondrial membrane integrity, releasing ROS and mitochondrial DNA, which trigger pro-inflammatory cytokines such as IL-18 and IL-1β, thereby promoting kidney cellular damage (36). Moreover, the NLRP3 inflammasome has been identified as a lipid-induced inflammatory mechanism. NLRP3 activation facilitates cytokines release and liver inflammation, and similar activation in renal tissues promotes immune responses (36, 37). Transcriptional regulators, particularly SREBP-1c and PPARα/γ, also play crucial roles in both diseases. SREBP-1c, a master regulator of lipogenesis, is persistently activated to promote hepatic lipid accumulation (38). Similarly, SREBP-1c activation leads to podocyte lipid deposition (39). In contrast, PPARα and PPARγ, which facilitate lipid clearance and exert anti-inflammatory effects, are typically downregulated in both diseases (40–43).

From transcriptomic analyses, we obtained 211 genes commonly associated with MN and NAFLD. Enrichment analysis indicated these genes were related to immune cells and cytokines activation. Hub gene screening revealed CSF1R as a key molecule through the random forest algorithm. In addition, immune infiltration revealed that CSF1R was correlated positively with monocytes in both diseases. Finally, scRNA-seq and immunohistochemistry were performed to validate our conclusions.

CSF1R is a receptor for both CSF-1 and IL-34, expressed primarily in macrophages, monocyte, microglia, osteoblasts, and myeloid dendritic cells (44, 45). It plays a key pro-inflammatory role and has been implicated in several kidney diseases, including acute kidney injury (AKI), lupus nephritis (LN), and focal segmental glomerulosclerosis (FSGS) (46–49). A study demonstrated that CSF1R is genetically increased in MN and promotes the expression of various cytokines (50). Multiple cytokines promote mesangial cell proliferation and alter hemodynamics leading to renal injury (51). Furthermore, the number of circulating monocytes has been shown to correlate with the severity of MN (52). Infiltrating monocytes in the kidney can differentiate into macrophages, which not only participate in tissue repair but also contribute to renal fibrosis (52–54). Monocytes may exert their damaging effects on the kidney through their surface receptor CSF1R. Our findings are consistent with previous studies. Therefore, we hypothesize that aberrant expression or functional dysregulation of CSF1R may initiate or exacerbate immune dysfunction in MN.

Our study also found an increase in CSF1R expression among patients with NAFLD. The biological functions of CSF1R are highly dependent on the activation of the CSF1/IL34–CSF1R signaling axis. In the context of NAFLD, this pathway appears to be upregulated, as indicated by elevated serum IL-34 levels and increased expression of the CSF1 gene (55, 56). Moreover, an increased expression of CSF1R was directly observed in the NAFLD animal model (57). CSF1R directly promotes lipid accumulation in hepatocytes via the glycolytic pathway (58). On the other hand, the CSF1/IL34–CSF1R signaling axis activates hepatic macrophages and produces pro-inflammatory cytokines, thereby promoting hepatic fibrosis (59, 60). Overall, CSF1R is a key molecule in the development and progression of NAFLD.

Our study has limitations. Due to its single-center design and relatively small sample size, a multi-center investigation is needed to further validate our findings. The cross-sectional study design made it difficult to infer a causal relationship between MN and NAFLD. Furthermore, animal experiments are needed to explore signaling pathways of CSF1R in both NAFLD and MN in greater detail. Patients with both diseases should be selected for validation, but such datasets are currently unavailable and should be carried out in the future.

## 5 Conclusion

In conclusion, we identify CSF1R as a shared molecular marker linking MN and NAFLD, implicating dysregulation of the monocytes in their co‐pathogenesis. Clinically, these findings suggest that regular monitoring of hepatic steatosis in MN patients may enable earlier detection of comorbidity. This study proposes a novel perspective that Chinese patients with MN are at increased risk of developing NAFLD, which may in turn exacerbate the progression of MN. Our results suggest that quantifying CSF1R expression may serve as a biomarker for the early and accurate diagnosis of patients with coexisting MN and NAFLD. In the future, targeting CSF1R signaling may represent a promising therapeutic strategy for mitigating both renal and hepatic injury.

## Data Availability

Publicly available datasets were analyzed in this study. This data can be found here: https://www.ncbi.nlm.nih.gov/geo/query/acc.cgi?acc=GSE1268482. https://www.ncbi.nlm.nih.gov/geo/query/acc.cgi?acc=GSE1973073. https://www.ncbi.nlm.nih.gov/geo/query/acc.cgi?acc=GSE896324. https://www.ncbi.nlm.nih.gov/geo/query/acc.cgi?acc=GSE1850515. https://www.ncbi.nlm.nih.gov/geo/query/acc.cgi?acc=GSE2008286. https://www.ncbi.nlm.nih.gov/geo/query/acc.cgi?acc=GSE1049487. https://www.ncbi.nlm.nih.gov/geo/query/acc.cgi?acc=GSE1295168. https://www.ncbi.nlm.nih.gov/geo/query/acc.cgi?acc=GSE2413029. https://www.ncbi.nlm.nih.gov/geo/query/acc.cgi?acc=GSE131685.

## Supplementary material

Supplementary Figure 1 Sample quality assessment to remove outlier samples. **(A)** Pearson correlation analysis was performed to calculate the correlation of gene expression between each sample of GSE197307. Samples with low correlation were removed as anomalous samples. **(B)** Visualization of pre-QC samples of GSE197307 using the PCA algorithm showed samples were clustered with each other. **(C)** Visualization of post-QC samples of GSE197307 using the PCA algorithm showed samples from different groups were separated. **(D)** Pearson correlation analysis was performed to calculate the correlation of gene expression between each sample of GSE126848. Samples with low correlation were removed as anomalous samples. **(E)** Visualization of pre-QC samples of GSE126848 using the PCA algorithm showed samples were clustered with each other. **(F)** Visualization of post-QC samples of GSE126848 using the PCA algorithm showed samples from different groups were separated.

Supplementary Figure 2 Performance evaluation of the model. **(A)** Confusion matrix of the RF model in the training set. Accuracy: 0.97, precision: 1.00, recall: 0.96, F1 score: 0.98. **(B)** ROC curve for the training set. The AUC was 0.94 (95% CI: 0.82–1.00). **(C)** Confusion matrix of the RF model in the test set. Accuracy: 0.92, precision: 0.86, recall: 1.00, F1 score: 0.92. **(D)** ROC curve for the test set. The AUC was 0.93 (95% CI: 0.79–1.00). RF, Random Forest; ROC, receiver operating characteristic; AUC, area under the curve.

Supplementary Figure 3 Representative immunofluorescence images of CSF1R (red) of renal and liver biopsies from HC, MN and NAFLD patients. Cell nuclei were counterstained with DAPI (blue). Scalebars, 100μm; MN, membranous nephropathy; NAFLD, non-alcoholic fatty liver disease.
