# Supplementary figures and images for: Identification of key genes in membranous nephropathy and non-alcoholic fatty liver disease by bioinformatics and machine learning
Source: Front Immunol. 2025 Jun 5;16:1564288. doi: 10.3389/fimmu.2025.1564288 (PMC12176592; doi:10.3389/fimmu.2025.1564288)

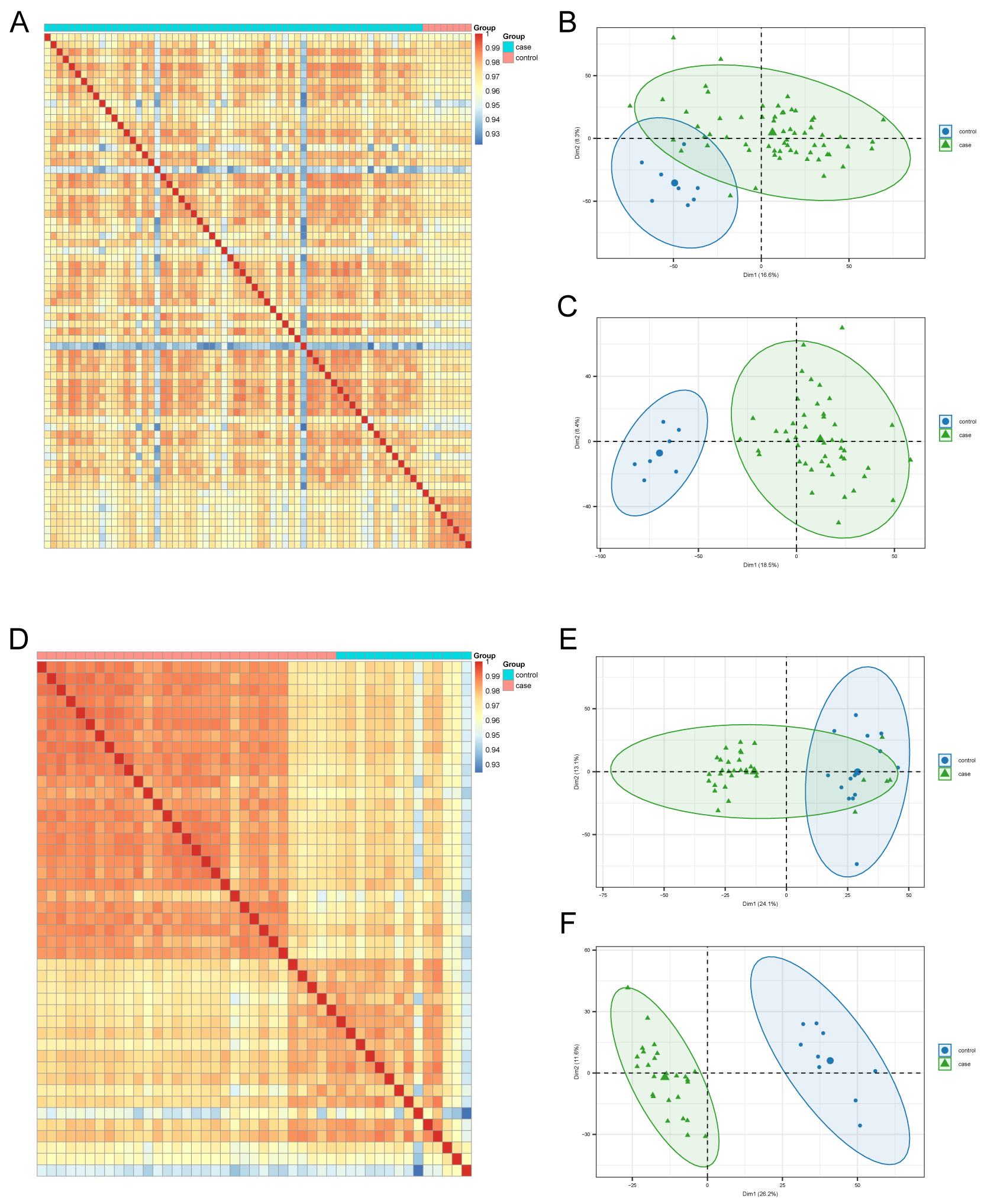

Supplement: Supplementary Figure 1 — Sample quality assessment to remove outlier samples. (A) Pearson correlation analysis was performed to calculate the correlation of gene expression between each sample of GSE197307. Samples with low correlation were removed as anomalous samples. (B) Visualization of pre-QC samples of GSE197307 using the PCA algorithm showed samples were clustered with each other. (C) Visualization of post-QC samples of GSE197307 using the PCA algorithm showed samples from different groups were separated. (D) Pearson correlation analysis was performed to calculate the correlation of gene expression between each sample of GSE126848. Samples with low correlation were removed as anomalous samples. (E) Visualization of pre-QC samples of GSE126848 using the PCA algorithm showed samples were clustered with each other. (F) Visualization of post-QC samples of GSE126848 using the PCA algorithm showed samples from different groups were separated. [file Image1.tif]

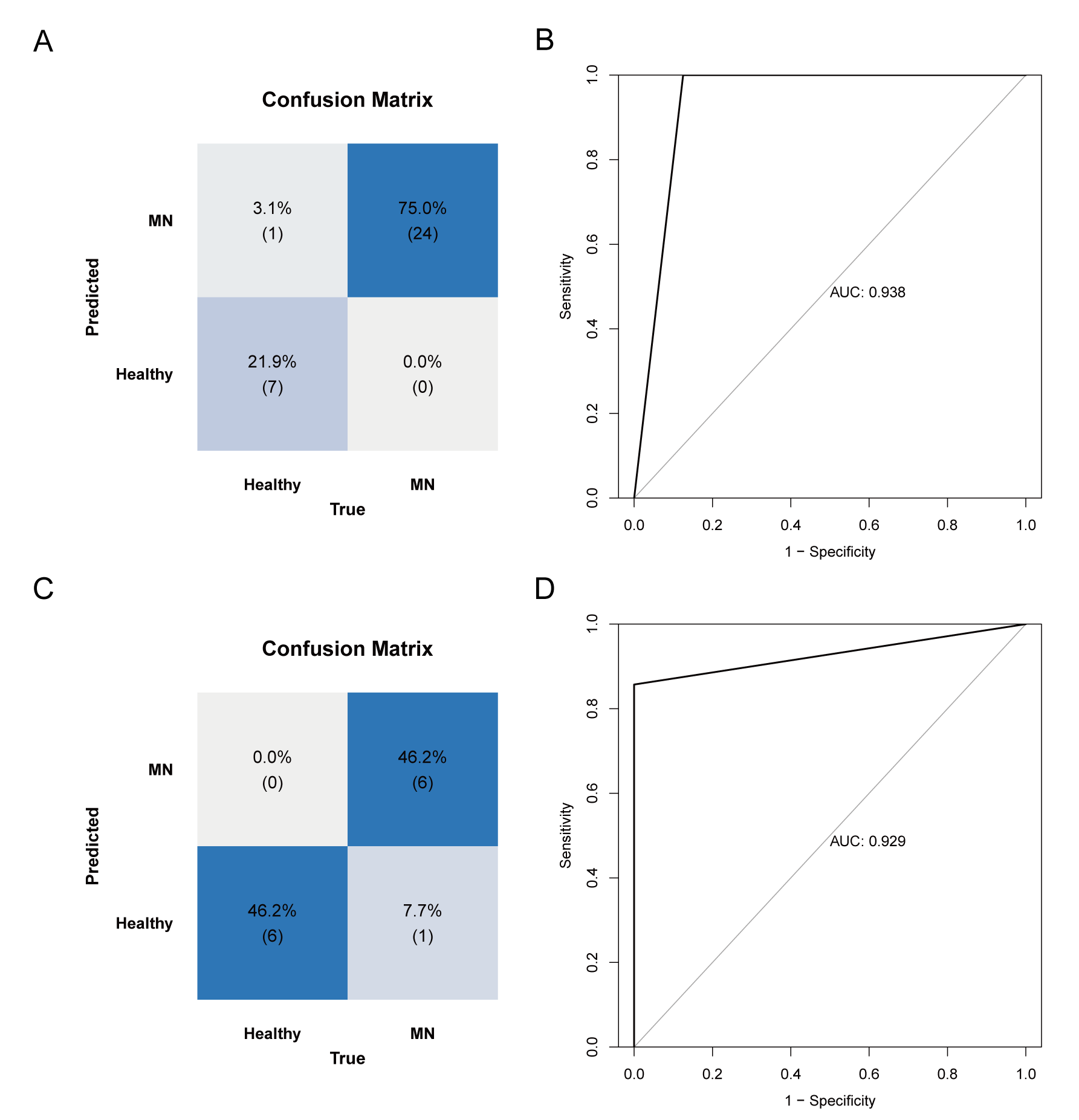

Supplement: Supplementary Figure 2 — Performance evaluation of the model. (A) Confusion matrix of the RF model in the training set. Accuracy: 0.97, precision: 1.00, recall: 0.96, F1 score: 0.98. (B) ROC curve for the training set. The AUC was 0.94 (95% CI: 0.82–1.00). (C) Confusion matrix of the RF model in the test set. Accuracy: 0.92, precision: 0.86, recall: 1.00, F1 score: 0.92. (D) ROC curve for the test set. The AUC was 0.93 (95% CI: 0.79–1.00). RF, Random Forest; ROC, receiver operating characteristic; AUC, area under the curve. [file Image2.tif]

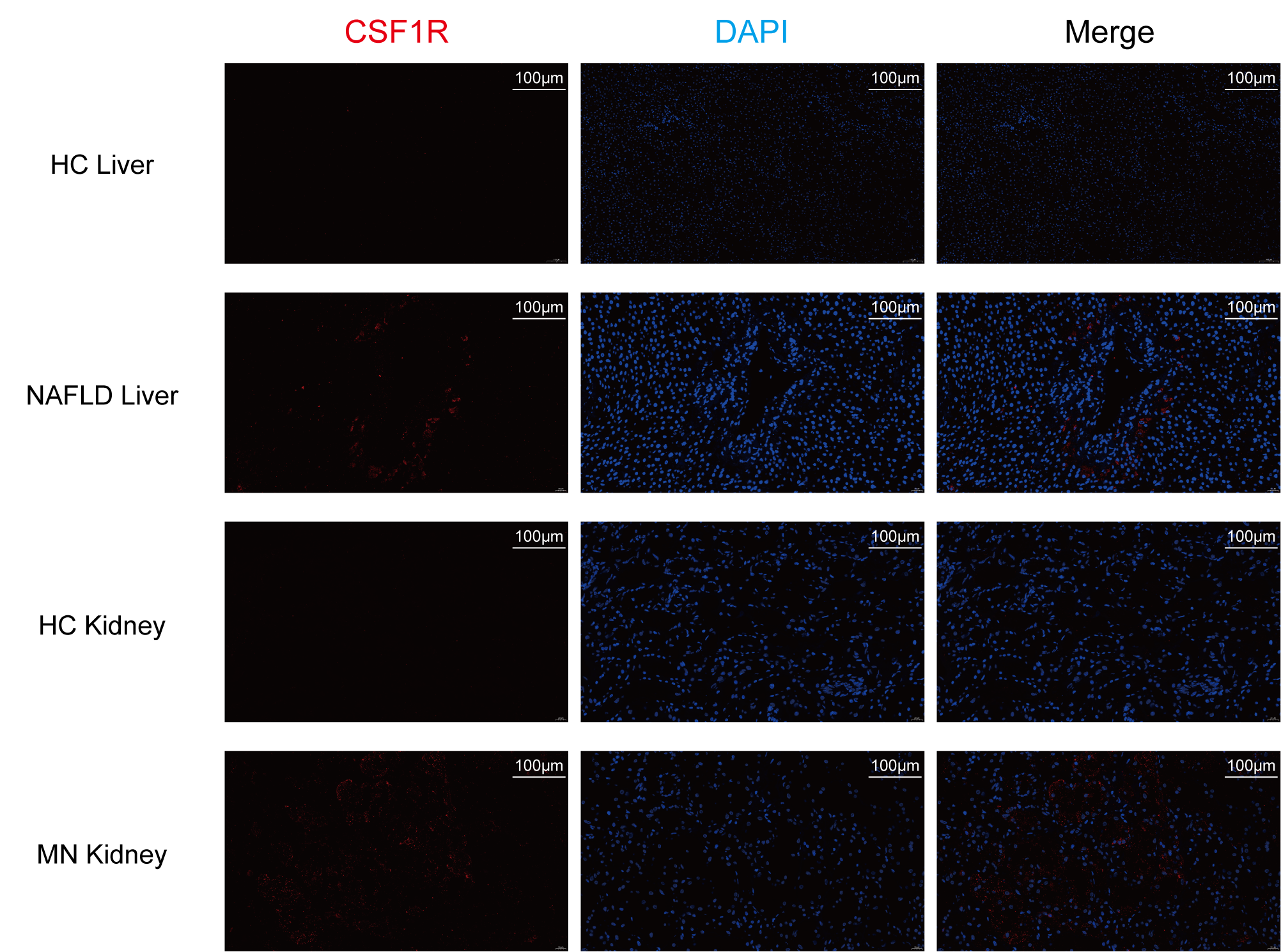

Supplement: Supplementary Figure 3 — Representative immunofluorescence images of CSF1R (red) of renal and liver biopsies from HC, MN and NAFLD patients. Cell nuclei were counterstained with DAPI (blue). Scalebars, 100μm; MN, membranous nephropathy; NAFLD, non-alcoholic fatty liver disease. [file Image3.tif]
