# Supplementary material for: Identification of key genes in membranous nephropathy and non-alcoholic fatty liver disease by bioinformatics and machine learning
Source: Front Immunol. 2025 Jun 5;16:1564288. doi: 10.3389/fimmu.2025.1564288 (PMC12176592; doi:10.3389/fimmu.2025.1564288)
Supplement: Supplementary file 4 [file Table1.docx]

Supplementary Table 1: Comparison of clinical information of MN patients in the NAFLD group and the non-NAFLD group

|  | NAFLD (n = 37) | non-NAFLD (n = 56) | *P* |
| --- | --- | --- | --- |
| Gender |  |  | 0.324 |
| Male (n, %) | 20 (54.1) | 36 (64.3) |  |
| Female (n, %) | 17 (45.9) | 20(35.7) |  |
| Age (years, $\bar{x}$±SD) | 52±12 | 49±14 | 0.356 |
| Height [cm, M (Q1, Q3)] | 167.00 (155.75, 170.00) | 163.62 (158.25, 168.88) | 0.434 |
| Weight (kg, $\bar{x}$±SD) | 75.02±12.75 | 66.69±10.28 | <0.001 |
| SBP (mmHg, $\bar{x}$±SD) | 137±18 | 135±21 | 0.535 |
| DBP (mmHg, $\bar{x}$±SD) | 82±13 | 77±11 | 0.083 |
| WBC [×10^^^9/L, M (Q1, Q3)] | 6.36 (5.55, 7.40) | 6.01 (4.98, 7.46) | 0.451 |
| Hb [g/L, M (Q1, Q3)] | 139 (127, 159) | 128 (111, 140) | 0.004 |
| PLT [×10^^^9/L, M (Q1, Q3)] | 254 (204, 351) | 239 (198, 288) | 0.274 |
| ALT [U/L, M (Q1, Q3)] | 19.80 (13.50, 27.20) | 16.60 (12.23, 31.65) | 0.502 |
| AST [U/L, M (Q1, Q3)] | 19.80 (16.75, 24.00) | 22.25 (17.23, 29.00) | 0123 |
| ALB (g/L, $\bar{x}$±SD) | 26.66±7.76 | 25.27±5.56 | 0.317 |
| CHOL [mmol/L, M (Q1, Q3)] | 6.77 (5.73, 9.38) | 6.81 (5.34, 8.31) | 0.524 |
| TG [mmol/L, M (Q1, Q3)] | 2.68(2.04,3.28) | 1.95(1.37,2.62) | 0.001 |
| HDL [mmol/L, M (Q1, Q3)] | 1.21 (1.09, 1.49) | 1.30 (1.16, 1.61) | 0.159 |
| LDL [mmol/L, M (Q1, Q3)] | 3.98 (3.19, 6.30) | 4.23 (3.33, 5.19) | 0.953 |
| BUN [mmol/L, M (Q1, Q3)] | 5.60 (3.92,6.52) | 5.45 (4.19,7.21) | 0.774 |
| Scr [μmol/L, M (Q1, Q3)] | 64 (47,73) | 62 (50,72) | 0.978 |
| UA [μmol/L, M (Q1, Q3)] | 381.76±95.06 | 351.71±98.94 | 0.143 |
| Cysc [mmol/L, M (Q1, Q3)] | 0.95 (0.88, 1.04) | 0.97 (0.83, 1.18) | 0.726 |
| GLU [mmol/L, M (Q1, Q3)] | 5.34 (4.51, 6.07) | 4.82 (4.43, 5.23) | 0.043 |
| 24h urine protein [g/L, M (Q1, Q3)] | 6.94 (3.56, 10.85) | 3.78 (2.56, 5.25) | 0.002 |
| PLA2R [RU/ml, M (Q1, Q3)] | 13.18 (3.40,126.31) | 15.16 (0,94.99) | 0.669 |
| IgA [g/L, M (Q1, Q3)] | 2.00 (1.39, 2.69) | 2.11 (1.46, 2.66) | 0.556 |
| IgG [g/L, M (Q1, Q3)] | 4.68 (3.44, 5.95) | 5.49 (4.36, 6.66) | 0.036 |
| IgM [g/L, M (Q1, Q3)] | 0.96 (0.68, 1.40) | 1.00 (0.73, 1.42) | 0.746 |
| C3 [g/L, M (Q1, Q3)] | 1.32 (1.19, 1.47) | 1.17 (1.00, 1.31) | <0.001 |
| C4 [g/L, M (Q1, Q3)] | 0.36 (0.28, 0.42) | 0.29 (0.25, 0.33) | 0.003 |
| D-Dimer [μg/ml, M (Q1, Q3)] | 0.65 (0.33,1.13) | 0.70 (0.34, 1.38) | 0.530 |
